# Neural Network Model of Urban Landscape Design Based on Multi-Target Detection

**DOI:** 10.1155/2022/9383273

**Published:** 2022-07-19

**Authors:** Fei Jia

**Affiliations:** Department of Fine Arts, Changzhi University, Changzhi 046011, Shanxi Province, China

## Abstract

Urban landscape design is of great significance to the development of the city. To solve the problem of manual acquisition characteristics of urban landscape design systems and low target detection accuracy, a new idea based on neural network and multi-objective testing technology construction urban landscape design system is proposed. By analyzing key technologies, the database establishment proposes to build a city landscape design system. Establish a city landscape design 3D model library using neural network and multi-objective testing technology. The system enables terrain measurements, GIS, visualization, massive data processing, virtual reality technology, etc., so that users can more and more effectively feel the rationality of space design and the feasibility of planning program. Through the experimental test, the following conclusions were obtained. First, the accuracy of multi-objective detection technology is maintained at around 88%. Second, the system landscape generation module generates fast, and the calculation time is between 0.57 and 46 s. Third, through analysis of the evaluation results of ecological suitability, the A-city landscape ecological function partition is divided into the plan for the plan to provide reliable data protection. The fourth is based on satisfaction evaluation indicators, which is conducive to the choice of the optimal plan of urban landscape design, thereby promoting the sustainable development of the city.

## 1. Introduction

With the continuous advancement of urbanization, urban landscape design is becoming more and more important. Urban landscape design is also an indispensable part of urban construction [1]. The development of information technology has promoted the huge change of urban landscape design. Using computer technology for planning and design can make urban landscape design more standardized and scientific.

Before the arrival of the digitization, the early landscape design is mainly based on language text and hand-painted renderings. Due to the multi-variability and complexity of urban landscape design in space and time, it is difficult to express the landscape design accurately [2]. Computer technology applies virtual reality technology to it, enabling people to conduct a full range of virtual landscapes in a virtual 3D environment. Compared with the traditional two-dimensional landscape design, the three-dimensional landscape design breaks through the two-dimensional description of the space, making the landscape design look more beautiful and real, saving a lot of time for designers. However, the landscape design system based on virtual reality technology is only designed to provide a three-dimensional virtual space for virtual landscape design, ignoring the relative position of virtual landscape and real scene, orientation, and size ratio. Traditional urban landscape design does not meet the requirements of scientific and high standards [3]. In fact, in the urban landscape design system, it is necessary to comprehensively use a variety of advanced technologies such as remote sensing and virtual simulation to do multi-scale, high resolution, multi-type, multi-time 3D description to achieve the city terrain landform, construction, road, transportation, garden, and other aspects of simulation, as well as objective reflection and digital recognition of the real situation of the city [4]. Therefore, in recent years, urban landscape is designed to become a research topic in the research community.

Target detection is the core technology of computer vision. The main research is the category we pay attention to, marking the goals from a frame in the image. It mainly includes two tasks: classification and positioning. Multi-target detection technology based on deep learning has become the mainstream of current target detection technology with the advantages of fast detection speed and high detection accuracy. In response to the standardization and scientific requirements of urban landscape design, this paper combines deep learning and multi-objective testing technology in urban landscape design, actively learns the inherent laws in the urban landscape database, and accurately describes the morphological characteristics by coding. The rules can save a lot of data collection and data analysis for designers and establish a more evidence-based design process. At the same time, deep learning and multi-target detection technology have realized the accurate detection of urban terrain, architecture, road, transportation, and gardens. The system analyzes different categories of space information in detail, providing strong decision-making support for urban distribution, building structure, garden design, time and space location, lighting, road, rendering, and noise, and achieves more scientific and reasonable urban landscape design and construction of beautiful cities.

## 2. Related Discussion

Urban landscape design is an application discipline across natural science and social science [5]. In these decades, with the development of technological progress and technology, especially in the promotion of digital technology as computer technology, the new landscape design and performance method began in people's field of view and developed rapidly. This has a certain impact on the traditional landscape design theory and practice.

The United States used a computer to complete the work of urban landscape planning, and the new technologies in urban landscape began application research. Foreign researchers try to increase their disciplines and architectural methods from urban planning and architecture demand from urban planning and architecture. Harvard University opened landscape urbanism, and digital technology was carried out in the landscape. They believe that the landscape must break through its aesthetics and symbol, increase the landscape ecological functionality, and use the city as an ecological community [6]. The University of London's Bill uses space syntax theory, with digital technology, to start urban landscape garden planning sectors [7]. In addition to digital technology conducted in college research institutions, some books such as “Bit City,” “digital survival,” and “future road” were published. It began to make people realize that digital technology changes people's lives, production and urban landscape planning, and design [8].

In addition, China also has some researchers and practitioners, providing a valuable reference for digital technology in the city of urban landscape design [9]For example,adopt AMAP to determine the basic model of plant structure in urban landscape design and simulate the growth of plants in a few years[10]; Li et al. analyzed the impact of waste heat from drainage on urban environment through Flu-net and Phonetics, and conducted environmental simulation analysis [11]. China National Geographic Center began building national map data, remote sensing images, geodetic libraries, special databases, and surveying and mapping data archives, especially the construction of spatial information libraries, providing more precise and convenient data for urban landscape planning and design [12]. The landscape designer also uses digital technology to create research and development, and the combination of landscape planning and digital technology has brought new opportunities for the innovation and change of urban landscape design. For example, Kim proposed the discussion of computer-assisted urban landscape modeling and provided methods for the modeling of landscape elements [13]. At the same time, it also discusses the application of CG technology in urban landscape planning and design, and puts forward the application of virtual reality technology in urban landscape planning and design [14]. In addition, with the cross-development of the discipline, the research results in other fields are referenced in the scenic garden discipline, and it has become a popular research direction. For example, Albawi et al. used convolution neural network for gesture recognition of RGB and RGB-D static images [15]. Venkatesan used radial basis function neural network for biometric iris recognition [16]. Tree-based convolution neural network was used to classify objects in segmented satellite images [17]. Hwang extracted R-CNN and wavelet feature for gesture recognition of EMG signal [18]. Tassinari proposed a research strategy study based on deep learning and explored the construction of urban landscape planning and design and landscape urbanism methodology as well as landscape planning design strategies for deep learning [19].

The target detection is the location and category information obtained from the image, and its primary task is to obtain the characteristics of the target. Relying on the maturity of image processing technology and the improvement of hardware equipment, the target detection algorithm is becoming more and more extensive in commercial applications. In 2012, a classification framework based on deep convolution neural networks and classification was first used in image classification by Krizhevsky. The accuracy rate has greatly improved compared to the traditional classification algorithm, laying the foundation for target testing research on deep learning. In 2014, Ross Girshick used exhaustion of the candidate box in the image to determine all possible areas and extracted the characteristics of the target in the candidate area, that is, a selective search algorithm was proposed. In 2015, Liu Wei proposed the end-to-end target detection algorithm. It was uniformly extracted from the candidate area, which greatly accelerated the detection speed and made deep learning applications more clear about the field of target detection.

With the popularization of intelligence, people increasingly want to get away from complicated work and devote themselves to more important work. Object detection is to simulate the process of human cognition and understanding of the world by using current information processing hardware and computer specific technology software. We can make use of the useful video image data detected by multi-object detection technology, which has an important role in promoting the development of current urban landscape design.

## 3. Urban Landscape Design System Construction

The essence of urban landscape design is to collect and store landscape information, and use relevant software and digital technology to realize the real landscape structure [20]. This part completes the task of urban landscape design through the overall module of urban landscape, the establishment of system database, and the practical application of the system.

### 3.1. System Overall Module

**Figure 1:**
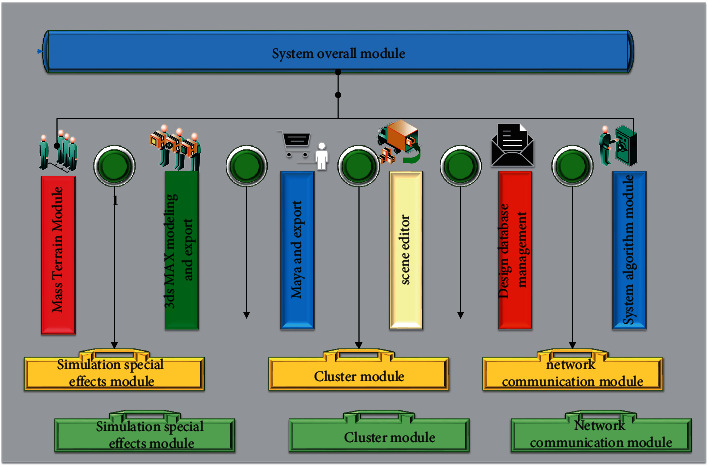
City landscape design system overall module map.

OSG is a high-level editable interface specially designed for 3D computing and graphics development and is widely used in virtual simulation, animation design, and various visualization programs. This system design proposes to build a city landscape design system based on OSG three-dimensional simulation support platform. Its functional goal is mainly to ensure that users can successfully master and relatively efficiently design and show relatively efficiently in a short period of time.  Figure 1 shows the overall module map of urban landscape design systems.

As shown in Figure 1, the large-scale terrain module is mainly established for urban landscape design, and the terrain is achieved as the amount of processing unit, and the numerical precision processing is provided. In the use of 3DS MAX modeling and output, Maya, and output modules, it is necessary to clarify the specific urban landscape design requirements and the architecture simulation scenarios, complete animation modeling software modifications, and export to the scene editor. In the Scene Editor module, the module can build a city landscape design scenario, which gives the original city landscape with simulation properties such as switch nodes, LOD, physical properties, collision, and other workflows.

Design the core working module, mainly as the core of OSG-based urban landscape design work, responsible for completing some core simulation tasks and completing the core graphics work, thereby achieving a collaborative work of the peripheral design module. It includes designing a database management module, a system algorithm module, and a variety of simulation modules. The system algorithm module includes deep learning and neural network algorithms, and the landscape scene design is the result of data processing by the system algorithm.

Cluster and network communication modules can directly connect the HLA host to implement remote off-site distribution, or use the protocol LAN to implement landscape simulation design. Not only can it significantly extend the calculation of virtual simulations, but also create a more reliable virtual environment, and to achieve efficient and fast network communication [21].

The multichannel visual interaction module is mainly to enhance the effect of urban landscape design and create a more open user operation vision [22]. By using the high-end display 360 ° three-dimensional ring screen technology to realize two channel and three channel simulation, the simulation cost is relatively high. Through the interaction of VR technology, users can really have an immersive experience.

### 3.2. System Database Is Established

#### 3.2.1. Entity Target Detection

In the process of urban terrain modeling, it is possible to combine different geographic object distribution characteristics, divided into discrete entities based on trees, roads, construction, etc., and continuity characteristics with urban terrain landform foundation. Use depth learning and multi-objective detection methods to complete the detection of object space.

**Figure 2:**
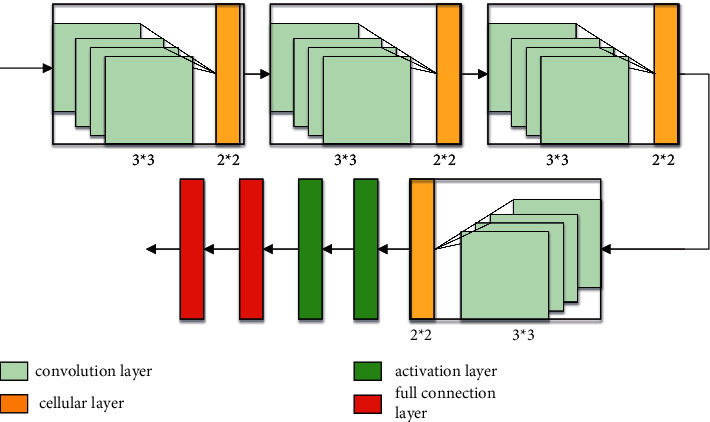
CNN structure schematic.

The development of multi-target detection technology has experienced the evolution from traditional multi-target detection technology to modern multi-target detection technology. The related technologies of modern multi-target detection mainly use more advanced technology to complete each step of traditional multi-target detection. This makes multi-target detection faster and more accurate. Multi-objective detection is to use deep learning technology for multi-objective detection. It is usually used by the machine to achieve a large number of artificial acquisition characteristics [23]. The characteristics of using deep learning network to extract candidate zone frames are the foundation of modern target detection technology, and it is also one of the most important steps of target detection and is related to overall time consumption and detection accuracy of target detection.  Figure 2 shows a typical CNN structure schematic, which is mainly composed of a convolution layer, a cellular layer, an activation layer, and a full connection layer.

As shown in Figure 2, the core portion of the convolution method in the CNN network is the consumption of the design and convolution layer of the convolution core. Convolution can be described as: given a function *d* (*k*), a point *k*, folding the function *d* (*k*) fold at the *k *point, and then traverses *k *for summing or integral. For continuous arguments, its calculation formula is

(1) $$\begin{array}{c} d\left(k\right)=b\left(k\right)\times c\left(k\right)=\prod b\left(x\right)*c\left(k+x\right)ex,\\ d\left(u\right)=b\left(u\right)\times c\left(u\right)=\overset{\infty }{\underset{x=-\infty }{∐}}b\left(x\right)*c\left(u+x\right). \end{array}$$

In the field of image processing, the convolution calculation of the city landscape design image is to slide on the image using the convolution nucleus or convolution template, multiplied by the pixel grayscale value on the image point, then add all multiplied values as the gradation value of the pixel corresponding to the intermediate pixel of the convolution, and finally slide all the pixel points. The formula is

(2) $$\begin{array}{c} z\left[u,v\right]=x\left[u,v\right]\times b\left[u,v\right]=\cup_{i}\sum_{i}b\left[u+i,v+j\right], \end{array}$$

where *u* and *v* represent the gray value of pixels, *i* and *j* represent the convolution nuclear value, and *z* [*u*, *v*] indicates the convolution calculation function.

**Figure 3:**
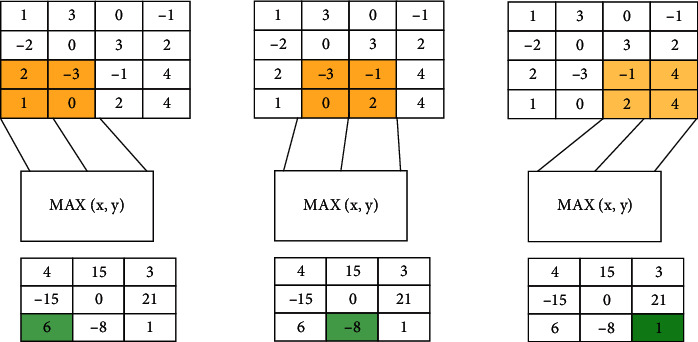
Image convolution calculation process.

The image convolution calculation process is shown in Figure 3.

The results of the entire city landscape design image volume can be clearly characterized by Figure 3. The picture uses a 3 ∗ 3 convolution. The 3 ∗ 3 convolution kernel can not only meet the requirements of image extraction but also improve the operation efficiency. The 3 ∗ 3 box size is taken in the image; then, multiply the pixel value of the output image with the corresponding pixel in the frame in the frame and obtain the output image. The use of the city landscape image processing is very broad, and image smoothing, blurring, drying, sharpening, and edge extraction can be accomplished by convolution operation.

#### 3.2.2. 3D Modeling

After completing the above image processing, the city landscape sets up a building group model library to explore, including construction, transportation, public facilities, buildings, plants, and three-dimensional model symbols such as green space, water, construction materials, and roads. The symbol library is combined to form a three-dimensional model. For most irregularities present in the actual city, by using transparent texture map technology, they can be textured, combined with two technologies, to obtain transparent and translucent irregularities.

#### 3.2.3. Database Is Established

**Figure 4:**
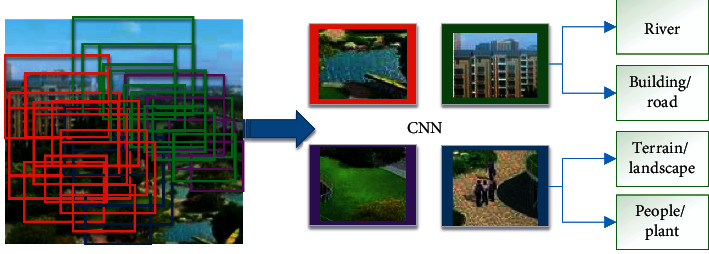
Database training process.

This design mainly uses SQL server technology to successfully establish a three-dimensional space database of urban landscape design systems. In order to test the influence of dataset pairs, it is trained for currently existing datasets and self-made datasets and compares different datasets on model accuracy. The training process is shown in Figure 4.

As shown in Figure 4, after training a network model, you need to select a standard, the loss function or the target function, and the resulting function or network performance [24].

(3) $$\begin{array}{c} T\left(f,m,n,p\right)=\frac{1}{N}\left(T_{\text{conf}}\left(f,m\right)-\varepsilon T_{\text{loc}}\left(f,n,p\right)\right), \end{array}$$

where *n* is the matching default box. If *N* = 0, Loss = 0. The definition of Smooth_L1_ is

(4) $$\begin{array}{c} {\text{Smooth}}_{L1}\left(f\right)=\left[\begin{array}{c} 0.5f^{2}-if\left|f\right|∠1\\ \left|f\right|+0.5-\text{otherwise} \end{array}\right). \end{array}$$

The definition of T_loc_ can be expressed as

(5) $$\begin{array}{c} T_{\text{loc}}\left(f,n,p\right)=f_{ij}^{k}{\text{Smooth}}_{T1}\left(n^{m}+p^{m}\right), \end{array}$$

where *T* is the prediction box, *f* is the group truth, (*f*, *n*, *p*) is the center of the default box *T* after compensation, (*i*, *j*) represent the width and height of the default box, *n* is a prediction box, and *P* is the ground truth.

(6) $$\begin{array}{c} T_{\text{conf}}\left(f,m\right)=\sum_{\text{iepos}}\text{log}\left(m_{i}^{p}\right)+\sum_{\text{ieneg}}\text{log}\left(m_{i}^{0}\right). \end{array}$$

Finally, test the network model on the test set obtained in the city landscape design. If the error is relatively large, the model will be equipped with the training set; if the error in the test set is small, then the model is very good.

### 3.3. System Application Function

**Figure 5:**
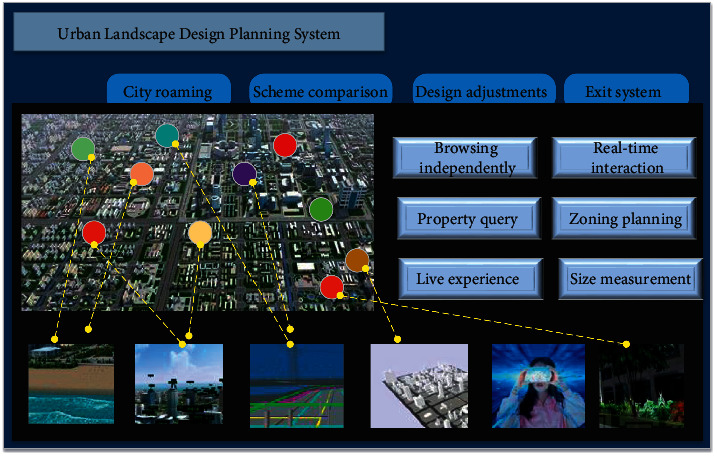
System application function diagram.

Through the three-dimensional urban landscape design system, it is possible to determine the volume, height, color, the relationship between the project and the relationship between the surrounding architecture and its sunshine spacing, and the stroke corridor, whether it meets the requirements of urban planning, thus understanding the city landscape. At the same time, the application of the system also solves the contradiction between the pursuit of interests and the planning department, as shown in Figure 5.

#### 3.3.1. Independent Browsing and Automatic Roaming

In the urban landscape design scene, users can control the walking route, and the walking mode can be walking, flying, etc. Users can explore the whole environment according to their own wishes, choose the content they want to experience, and can also rise and fall at any time. The observation angle is unlimited, and multiple observation points can be replaced. The operation is simple and fast. At the same time, users can also develop their own tourist routes. The number of lenses is controlled by the user and can be switched at any time.

#### 3.3.2. Real-Time Interaction and Attribute Queries

At some point, users may need to perform real-time editing in the objects in the city landscape design scenario and compare the effects reflected in different designs, such as program contrast, building height adjustment, and light and shadow analysis. Get attribute acquisition in the scene is also one of the interactions of the user, such as querying a building, where the company is located or the location and status information of hydro power, communication devices.

#### 3.3.3. Partition Planning and Size Measurement

This feature is very wide in urban landscape design. You can divide the entire city into several areas; each area is expressed in different colors, which can also classify different functions, such as residential area, commodity area, and planning area. Make each user more in the planning and future development of the city. Size measuring is a tool in a digital urban system, which can achieve distance measurement, area measurement, and different object volume measurements. Users can always understand the area of each piece of land and distance from the city center. This provides an accurate and fast tool for the digital urban system.

#### 3.3.4. Dance Experience

The display method of virtual reality can be a normal computer display or a flat or loop projection. With stereoscopic glasses, users can also see a very strong picture effect [25]. Imagine that in such a scene, you can use your own will or stop watching, and this experience is an effect picture, and animation and sandbox cannot be achieved. With the steering wheel, the user can simulate driving in the scene while simultaneously controlling the speed rhythm of driving. With a ring curtain projection system, all people are integrated into the scenes, and there is a feeling of impassiveness.

## 4. Experimental Design

### 4.1. Training Network Hardware Environment

Deep study and multi-objective testing urban landscape design training network model, involving a large number of matrices parallel operations and floating point calculations, need to consume a lot of computing resources. GPU relies on its super-kernel and trillion floating point operations, and it has become a must-have tool for deep learning research [26]. Therefore, the quality of GPU computer performance directly affects the efficiency and accuracy of model training. The hardware resources used in this experiment are shown in Table 1.

### 4.2. Evaluation Indicator

Deep learning and multi-objective testing are essentially classified for the goal of urban landscape design scenarios, and its purpose is to construct a classification function or classification model that maps the data object to a given category through the classifier. Common model evaluation indicators include precision rate, recall rate, and accuracy. In Table 2, TN refers to the number of negative samples correctly predicted; FN represents the number of negative spiked negatives; FP is the number of positive samples predicted; and TP is the right sample number of correctly predicted.

From the above four indicators, three common evaluation classifier performance can be obtained.

(7) $$\begin{array}{c} \text{Precision}=\frac{\text{TP}}{\left(\text{TP}+\text{FP}\right)}\text{,}\\ \text{recall}=\frac{\text{TP}}{\left(\text{TP}+\text{FN}\right)}\text{,}\\ \text{accuracy}=\frac{\left(\text{TP}+\text{TN}\right)}{\left(\text{TP}+\text{TN}+\text{FP}+\text{FN}\right)}. \end{array}$$

Among them, precision is also known as the ratio, which is the proportion of true and positive samples in the positive sample predicted by the classifier. Recall is also known as the full rate, and it refers to the number of classifiers found in all true and positive samples. Accuracy is a total ratio of the classifier to the overall judgment of the model, that is, the total proportion of the correct predictive target. In the experiment, the training loss value is also required, which refers to the distance between the real value of the problem and the model prediction value.

### 4.3. Establishment of Datasets

In the field of computer city landscape design research, image dataset plays a vital role. The dataset in this article includes 300,000 images, 2000,000 instances, and 100,000 key points, and each image contains 5 subtitles and 80 types of objects. The document of its dataset is detailed and has a special team maintenance. Most researchers use it as a “inspection” performance dataset in the field of image detection algorithms.

## 5. Results and Analysis

### 5.1. Multi-Objective Test Training

In the city landscape design, the annotation of 4 datasets was conducted through the expansion and training of datasets. The main improvements of each of them are as follows: SSMCITY1 mainly selects a small amount of target river classes in the universal dataset for labeling; SSMCITY2 is fully tagged for the target building and the road to the sample; SSMCITY3 modifies the marked sample, the terrain, and green scene for fine labeling; and SSMCITY4 increases sample richness and fineness, such as marking vegetation, pedestrians, and so on. The sample quantity of each labeled dataset under different perspectives is shown in Table 3.

Comparison can see the minimum amount of samples under the side perspective, and with the improvement of the target richness and fineness, the sample size detection of urban landscape design targets is more and more samples from the front view, side view, and back angles.

**Figure 6:**
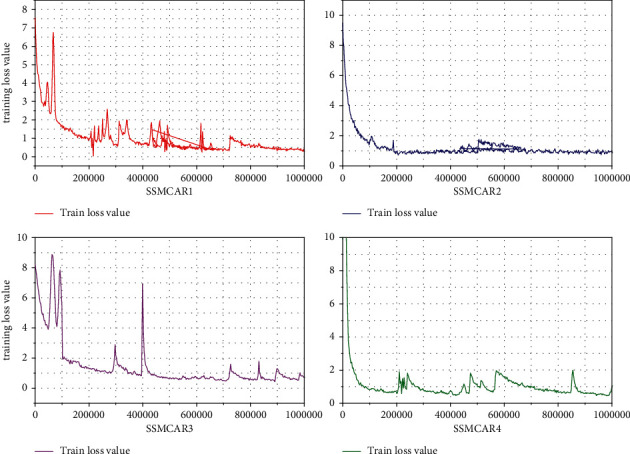
Loss curve of online training.

The SSD target detection algorithm is used. The basic network model uses VGG16. The image of the input network is adjusted to a fixed 1024 ∗ 1024 size, and the number of iterations of training is set to 120000 times, and training and testing are carried out for the created dataset. The loss curve of network training is shown in Figure 6.

As can be seen from Figure 6, the loss gradually decreases as the number of iterations is increased, and when the loss value is substantially reduced to 1 after iteration 120000, it explains the convergence requirements. Due to the influence of GPU performance, the batch size is set to 4 when the loss curve is swayed.

**Figure 7:**
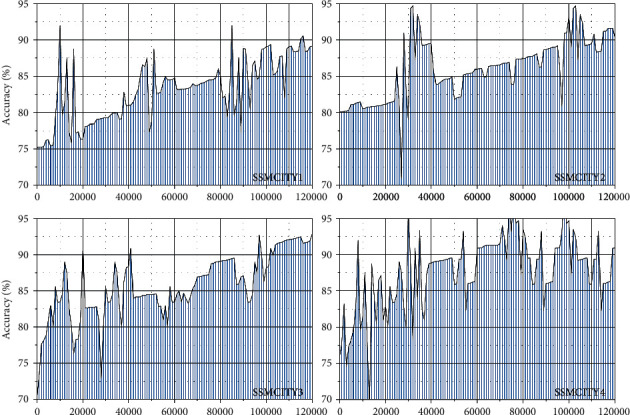
Accuracy curve of network training.

By testing different datasets, as shown in Figure 7, it can be found that accuracy has gradually increased, iterates to a certain number of times, and tends to change gently; after each improvement of the sample of the dataset, the accuracy value is not too large, and the average detection accuracy is basically around 88%, but its loss value is getting smaller and smaller, and the network will detect stability and confidence.

Through the training of different datasets, the training model of its different output results is obtained.  Table 4 shows the number of training samples of several datasets and analysis accuracy analysis. It can be seen from the table that the target detection accuracy is the highest, and the pedestrian target detection accuracy is the lowest, mainly due to too small pedestrian targets in the scene. The average detection of the SSMCAR4 dataset has reached 89.5%.

### 5.2. Landscape Generation Test

**Figure 8:**
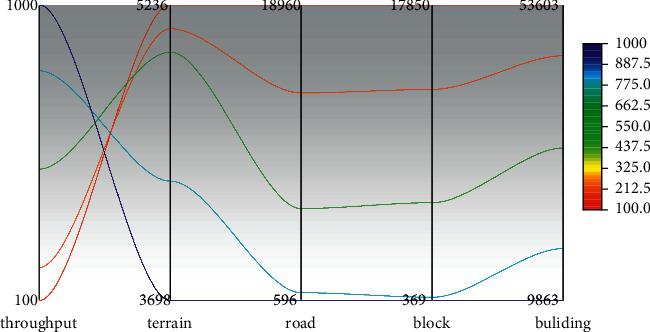
City landscape design generation module time test.

According to the module used in urban landscape design generation, the system test project is divided into four stages, the terrain map generation phase, the road model generation phase, the block landscape phase, and the architectural phase. A total of three test cases have been included to evaluate the system. For each test case, it will be tested 10 times and the average of the results will be taken to get more accurate results, as shown in Figure 8.

It is seen from Figure 8 that by a lateral comparison, it can be seen that the more the number of processes in each frame, the faster the system speeds. However, if there is too much entity in one time, the frame rate at the runtime will fall. Therefore, it is found that the balance between the calculation time and the frame rate can be another topic. Although most of the time is spent on the architectural phase, in general, the calculation time is between 0.57 and 46 s, within an acceptable range.

### 5.3. Ecological Maturity Evaluation

**Figure 9:**
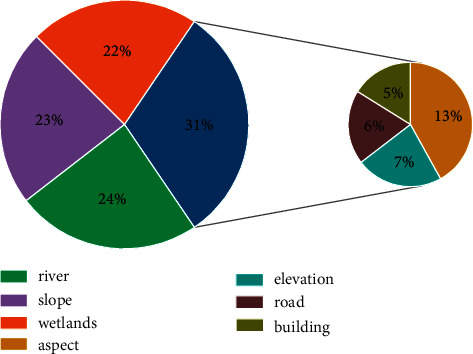
Ecological suitability evaluation index.

Ecological suitability analysis is an evaluation and analysis of the combination of urban environmental status and construction development demand and then reasonably estimates the problems that may occur after construction, in order to reflect the ecological suitability and development potential of research site [27]. Coordinate the design characteristics of urban landscape design based on the evaluation factors in the ecological suitability analysis. Through the means of on-site surveys, expert discussion, data research, etc., ultimately determine slopes, slope, elevation, roads, rivers, gardens, buildings, etc. to establish an ecological suitability evaluation system, as shown in Figure 9.

As can be seen from Figure 9, in the influence of urban landscape design evaluation factor weights, the river is the factor that affects the effects of regional ecological suitability evaluation, which has a great impact on the landscape design of the research area. The weight value of the slope factor is slightly lower than the former, and it is also a key element of ecological suitability evaluation. The remaining evaluation factor weights are sequentially decremented by slope factors, elevation factors, road factors, and building factors and decrease in ecological suitability evaluations, but there is also an important role in landscape planning.

**Figure 10:**
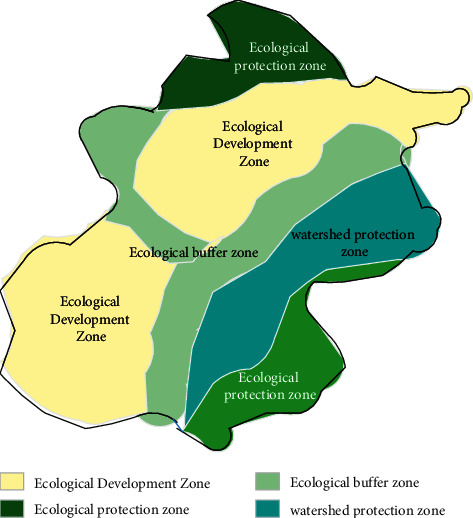
A city landscape ecosystem partition.

As shown in Figure 10, the research area is divided into four suitability levels in the ecological suitability evaluation of A, which is not suitable, low, high, and optimum. Inappropriate area, there is a steep slope, close to the water, vegetation is rich, and has high ecological sensitivity, not suitable for development and construction, need to make ecological protection; low-appropriate development zones should choose protection or low degree according to the actual situation Development. By analyzing the evaluation results of the ecological suitability, a city landscape ecological function partition division is divided into this geographical spatial partition result. A city landscape ecological function partition is divided into four types of ecological functional zones: ecological protected areas, basin protection areas, ecological buffers, and ecological development zones.

### 5.4. Landscape Design Satisfaction Evaluation

Urban landscape design is related to the importance of urban future development. Urban landscape design needs to follow the requirements of “meeting the public, reflecting the local, adapting to the times, beautiful, economical, and applicable.” According to the evaluation results, the evaluation of urban landscape design can be divided into six categories. This is the evaluation, technical assessment, comprehensive assessment, landscape aesthetic quality evaluation, landscape value evaluation, and landscape impact assessment. The specific evaluation indicators are shown in Table 5.

**Figure 11:**
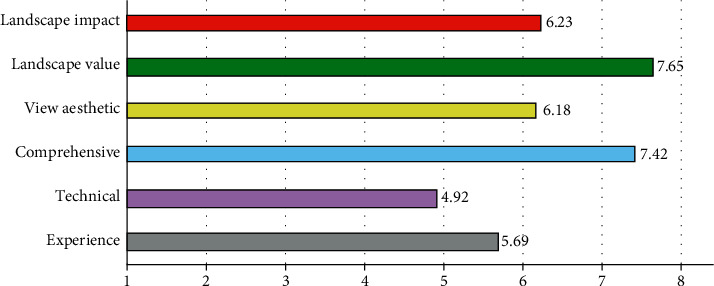
Evaluation index weight.

According to the above evaluation indicators, an evaluation index system is established. Randomly find 150 evaluation subjects for questionnaire survey, and the evaluation subjects are divided into 20 experts, 30 designers, and 100 citizens. Then, the questionnaires were counted to determine the weight of each evaluation index. Finally, the evaluation scores of each factor are comprehensively processed to obtain a comprehensive evaluation score. The evaluation indicator weight is shown in Figure 11.

As can be seen from Figure 11, the maximum indicator weight is the landscape value evaluation and comprehensive assessment, and the weight is 7.65 and 7.42, respectively. Second is the landscape impact assessment and landscape aesthetic quality evaluation, and the weight is 6.23 and 6.18, respectively; The last category is experiential evaluation and technical evaluation, with weights of 5.69 and 4.92, respectively. According to the evaluation indicators at all levels, choose the optimal plan of urban landscape design, which is conducive to the optimization of the landscape and the sustainable development of the city.

## 6. Conclusion

Urban landscape design is an indispensable part of urban construction development. The establishment of the urban landscape design system based on deep learning and multi-target testing has achieved accurate testing of multiple targets such as urban terrain, architecture, roads, transportation, and gardens. The target testing of buildings and road categories is the highest, while pedestrian target detection accuracy is the lowest, and the average detection accuracy is basically around 88%. The loss value has basically decreased below 1 after iteration of 120,000 times, indicating that it meets the requirements of convergence. By analyzing different types of spatial information, the system can provide people with various realistic scene designs about the urban landscape in an all-round and intuitive manner and build a real and intuitive virtual urban landscape environment. Through systematic ecological suitability evaluation and systematic satisfaction evaluation, the urban landscape space design can be more scientifically and rationally carried out, and the feasibility of the planning scheme can be evaluated, so that the future urban landscape can be more suitable for people's aesthetic needs, so as to beautify people's living environment and promote the health of the city and sustainable development. Deep learning and multi-object detection techniques have developed rapidly in urban landscape design applications, especially in detection and recognition tasks. With the coordinated development of cities and rural areas, whether the urban landscape design system of this paper can be extended to rural landscape design is a direction that needs further research in the future.

## Figures and Tables

**Table 1:** Hardware resources.

Hardware type	Equipment 1 (initial use)	Equipment 2 (post development)
GPU model	NVIDIA TITANX	NVIDIA 1080TI
GPU number	2	1
CPU model	Intel(R) Core(TM) i7-6800k	Intel(R) Core(TM) i7-6800k
ROM	64G DDR3	32G DDR3
RAM	1 TB SSD + 5 TB mechanical hard disk	1 TB SSD + 2 TB mechanical hard disk

**Table 2:** Model evaluation index.

Predicting category	Indicator content
TN	Correctly predicted negative samples
FN	Indicates the number of negative predictions
FP	The number of positive samples that are wrongly forecast
TP	Correctly predicted number of active samples

**Table 3:** Sample amount below different perspectives.

Dataset name	SSMCITY1	SSMCITY12	SSMCITY3	SSMCITY14
Front view	2049	2293	3053	6789
Side view	537	554	635	1465
Rear perspective	1984	2045	2899	4562

**Table 4:** Dataset detection accuracy analysis.

Dataset name	SSMCITY1	SSMCITY12	SSMCITY3	SSMCITY14
Total sample	5000	6127	7186	10186
Training verification	4499	5514	6167	9165
Test set	501	613	719	1021
River	0.866	0.862	0.86	0.88
Building/road	0.877	0.886	0.89	0.92
Terrain/landscape	0.888	0.899	0.9	0.899
People/plant	—	—	0.884	0.884
Total accuracy	0.877	0.881	0.884	0.894

**Table 5:** City landscape evaluation index.

Evaluation indicator	Evaluation standard
Experience evaluation	The public evaluation of the quality of urban landscape
Technical assessment	Evaluation of the appropriate level of the subject in urban life
Comprehensive assessment	City landscape resource overall value evaluation
View aesthetic quality evaluation	The beauty of the landscape passenger form
Landscape value evaluation	Urban landscape resource, economical evaluation
Landscape impact assessment	Keep the sustainability of the landscape

## Data Availability

The data used to support the findings of this study are included within the article.
